# SCO‑792 alleviates HFD‑aggravated chronic pancreatitis by modulating the gut–pancreas axis via the AMPK/ACC and TLR4/NF‑κB signaling pathways

**DOI:** 10.3389/fimmu.2026.1923107

**Published:** 2026-09-03

**Authors:** Yanru Kang, Ningning Sun, Weijie Yao, Jinyu Li, Hua Yin, Jiyue Wang, Zuozheng Wang, Shaoqi Yang, Xiaoli Yang

**Affiliations:** 1 Department of Gastroenterology, General Hospital of Ningxia Medical University, Yinchuan, China; 2 Department of Hepatobiliary Surgery, General Hospital of Ningxia Medical University, Yinchuan, China

**Keywords:** SCO-792, chronic pancreatitis, gut–pancreas axis, AMPK/ACC, TLR4/NF-κB

## Abstract

**Background:**

Chronic pancreatitis (CP) is an inflammatory disorder that manifests as pancreatic fibrosis and exocrine insufficiency, and premature activation of trypsinogen to trypsin. Enteropeptidase has emerged as a promising therapeutic target in CP due to its critical role in activating trypsinogen. The aim of this study was to assess the effects of SCO-792, a novel enteropeptidase inhibitor, on high-fat diet (HFD)-aggravated CP, and explore the underlying mechanisms.

**Methods:**

A mouse model of HFD-aggravated CP was established by chronic caerulein administration. The protective effects of SCO-792 were evaluated through histological analysis, biochemical assays, and molecular techniques. The composition of the gut microbiota was analyzed by 16S rRNA gene sequencing, and the fecal levels of short-chain fatty acids (SCFAs) were measured. The involvement of the gut-pancreas axis was further validated by supplementation with *Akkermansia muciniphila* (AKK) and propionate.

**Results:**

SCO-792 treatment significantly alleviated HFD-aggravated pancreatic injury and fibrosis, as indicated by reduced histological scores, decreased collagen deposition, and downregulated protein levels of collagen I and α‑SMA. At the metabolic level, SCO-792 regulated pancreatic lipid metabolism homeostasis via the AMPK/ACC pathway. SCO-792 also restored the gut microbiota by increasing the abundance of *Akkermansia* and other beneficial bacteria, and enhancing production of SCFAs. Furthermore, SCO-792 significantly reduced the translocation of serum lipopolysaccharide(LPS) by maintaining intestinal barrier integrity. The decrease in systemic endotoxin levels eventually blocked the TLR4/NF-κB-mediated inflammatory response in the pancreas, and promoted macrophage polarization to the M2 phenotype. Supplementation with *Akkermansia* or propionate protected against HFD-induced CP through similar signaling pathways, and synergistically activated AMPK signaling while suppressing inflammatory pathways.

**Conclusion:**

SCO-792 mitigated HFD-aggravated pancreatitis in mice by modulating the gut-pancreas axis, indicating its potential as a preventive and therapeutic agent for metabolic pancreatic diseases.

## 1 Introduction

Chronic pancreatitis (CP) is a progressive fibroinflammatory disease affecting the pancreatic parenchyma and ductal system, which eventually progresses to exocrine and endocrine insufficiency, and can severely impair quality of life and increase mortality risk (1, 2). The global incidence of CP in adults ranges from 5 to 12/100,000, and epidemiological studies indicate a continuously rising trend (3). The pathogenesis of CP involves a complex interplay of genetic susceptibility, aberrant immune responses, and environmental factors (4). In recent years, high-fat diet (HFD) and obesity-related metabolic disturbances have been recognized as key drivers of CP. Long-term high-fat intake not only induces acinar cell lipotoxicity, but also accelerates pancreatic fibrosis by increasing oxidative stress and endoplasmic reticulum stress (5, 6). Furthermore, hypertriglyceridemia is closely associated with recurrent episodes of acute pancreatitis (AP), and its transition to the chronic stage (7, 8). At the cellular level, the core initiating event in pancreatic injury is the premature, aberrant activation of trypsinogen within acinar cells (9). Under physiological conditions, trypsinogen is synthesized in its inactive form and stored in zymogen granules within acinar cells. Upon entry into the intestine, it is activated by enterokinase, thereby initiating the cascade activation of other digestive zymogens (10). However, sustained HFD consumption can trigger premature activation of trypsinogen within acinar cells (11), leading to cellular autodigestion and necrosis. In addition, the damage-associated molecular patterns (DAMPs) released from these necrotic cells can activate the resident innate immune cells, potentially inducing both local and systemic inflammation (12).This inflammatory cascade features dysregulated secretion of pro-inflammatory cytokines including TNF-α, IL-1β and IL-6, whereas the anti-inflammatory cytokine IL-10 restrains excessive inflammatory activation.

Enteropeptidase mediates physiological trypsinogen activation in the intestinal tract (13). Pathological premature trypsinogen activation in pancreatitis mainly occurs within pancreatic acinar cells and is largely independent of intestinal enteropeptidase. Our previous work demonstrated that enteropeptidase initiates the activation of trypsinogen at the very origin in the intestine, which subsequently triggers a downstream trypsinogen-dependent enzymatic cascade (14, 15). SCO-792, an enteropeptidase inhibitor, can block trypsinogen activation and suppress the downstream pancreatic enzyme cascade (16), and has been shown to prevent the occurrence of post-endoscopic retrograde cholangiopancreatography pancreatitis (PEP) (17). In addition, SCO-792 exerts protective effects in animal models of metabolic syndrome and obesity by modulating the intestinal microflora and regulating production of microbial metabolites, such as short-chain fatty acids (SCFAs) and bile acids (18). Furthermore, SCO-792 enhances insulin sensitivity and improves blood glucose levels in mouse models of diabetes (19). Notably, SCO-792 may also affect intestinal protein digestion, nutrient absorption and systemic energy metabolism, which may mediate its protective effects observed in diabetic patients and chronic kidney disease rats (20, 21).Nevertheless, the effects of SCO-792 in chronic pancreatitis have not been elucidated so far.

Recent evidence has implicated the gut microbiota in the pathogenesis of CP. Compared to healthy individuals, CP patients exhibit a mild decrease in the α-diversity of gut microbes, along with significantly altered β-diversity. Specifically, the abundance of Firmicutes, and beneficial genera such as *Coprococcus* and *Bifidobacterium* is reduced, while that of potentially pathogenic genera within the Enterobacteriaceae family, such as *Escherichia-Shigella* and *Klebsiella*, is increased in CP patients (22). Furthermore, intestinal dysbiosis can directly induce fibro-inflammatory changes in the pancreas (23). For instance, SCFA-producing Gram positive bacteria, such as *Roseburia* and *Faecalibacterium*, are markedly reduced in CP patients and mouse models. Exogenous supplementation with SCFAs has been shown to effectively alleviate pancreatic fibrosis, with the mechanism involving the inhibition of macrophage infiltration and their polarization toward the M2 phenotype (24). These findings suggest that the depletion of SCFA-producing bacteria may be an important driver of the fibrotic process in CP. In addition, an impaired intestinal barrier is closely associated with metabolic disturbances. Increased fat intake increases intestinal permeability, leading to metabolic endotoxemia, which in turn promotes pancreatic cancer development and pancreatic fibrosis (25). Therefore, the gut microbiota has gained attention as a viable therapeutic target for CP.

The aim of this study was to evaluate the therapeutic effects of SCO-792 in a mouse model of HFD-aggravated CP, and explore the involvement of the gut-pancreas axis. Our findings provide new insights into the pathogenesis and progression of CP associated with high fat intake, and establish an experimental basis for the preventive and therapeutic application of SCO-792.

## 2 Materials and methods

### 2.1 Animals

Six-to-eight weeks old male C57BL/6 mice (Ningxia Medical University) were housed in a specific pathogen-free facility under ambient conditions (23 ± 2 °C, 44%-52% relative humidity) and a 12 h light/dark cycle. After one week of adaptive feeding, the mice were randomly assigned to the normal control (NC), HFD, caerulein (CAE) and SCO-792 groups according to body weight, with 6–8 mice per experimental group. The mice in the NC group received the regular feed, and those in the remaining groups were fed HFD for four weeks. CP was induced by the intraperitoneal injection of CAE (MedChemExpress [MCE], Cat. No. HY-A0190; 50 μg/kg, 8 injections per day at 1-hour intervals). CAE was administered three days per week, with a one-day interval between injection days for a total of four weeks. SCO-792 (MCE, Cat. No. HY-132841A) was administered daily through oral gavage at the dose of 50 mg/kg; this dosage was referenced from previous reports (19–21); the mice in the NC and HFD groups received the same volume of sterile normal saline. In a separate experiment that was conducted to validate the role of gut microbiota, the mice were administered an antibiotic cocktail (0.5 g/L vancomycin [V2002, Sigma-Aldrich], 1 g/L neomycin [N6386, Sigma-Aldrich], 1 g/L metronidazole [M3761, Sigma-Aldrich], and 1 g/L ampicillin [A0166, Sigma-Aldrich]) in drinking water for one week, and then randomly assigned into the NC, HFD, CAE, *A. muciniphila* (AKK), sodium propionate (PA), and AKK+PA groups. CP was induced in all HFD-fed groups (except NC) using the same CAE protocol. Concurrently, AKK (1×10^9^ CFU/mouse/day) and/or PA (150 mM/day) was given by oral gavage for 4 weeks; the NC and HFD groups received sterile normal saline. After five weeks (antibiotic pre-treatment plus HFD feeding and CP induction with interventions), the mice were euthanized by carbon dioxide (CO_2_) inhalation at the end of the experiment, and blood, pancreas, ileum, and colon tissues were harvested and immediately stored at −80 °C. All mice had ad libitum access to food and water. Mice in the NC group were fed Regular Chow Diets (05073, Ningxia Medical Laboratory Animal Center), whereas the remaining groups received a high-fat diet (HFD; 60% kcal fat, Cat. No. MD12033, Medicience). The animal experiments were approved by the Animal Ethics Committee of Ningxia Medical University (Approval number: IACUC-NYLAC-2024-048).

### 2.2 Culture of *A. muciniphila*

*A. muciniphila* (ATCCBAA-835) was first activated on ATCC Medium 260 (Tryptic Soy Medium with 5% Defibrinated Sheep Blood) agar plates under anaerobic conditions. The activated *A. muciniphila* strain was then inoculated into liquid medium with a 1% (v/v) inoculum and incubated statically at 37 °C for 72–96 h under anaerobic conditions. Bacterial cells were collected via centrifugation (8,000 × rpm, 10 min, 4 °C). The cell pellet was washed twice with sterile normal saline (0.9% NaCl) and subsequently resuspended in sterile normal saline for oral administration to mice.

### 2.3 Biochemical tests

Assay kits for triglycerides (TG, Cat. No. A110-1-1), high-density lipoprotein cholesterol (HDL-C, Cat. No. A112-1-1), low-density lipoprotein cholesterol (LDL-C, Cat. No. A113-1-1), and total cholesterol (TC, Cat. No. A111-1-1) were purchased from Nanjing Jiancheng Bioengineering Institute. Serum levels of the lipids were measured as per the kit instructions.

### 2.4 Fecal elastase activity measurement

Fecal elastase activity was determined using the EnzChek™ Elastase Assay Kit (Thermo Fisher Scientific, catalog E12056) according to the manufacturer’s protocol. Briefly, frozen fecal samples were homogenized in assay buffer, centrifuged, and the supernatants were incubated with DQ-elastin substrate. Fluorescence intensity was measured at Ex/Em = 505 nm/515 nm. Porcine pancreatic elastase was used to generate a standard curve. Fecal elastase activity was normalized to fecal wet weight and expressed as relative fluorescence units (RFU)/mg feces.

### 2.5 Histological assessment

Pancreatic and intestinal tissue samples were fixed in 4% paraformaldehyde, dehydrated with graded ethanol, embedded in paraffin, and cut into 5 μm-thick sections. Hematoxylin and eosin (H&E) staining was performed as per standard protocols, and the stained sections were viewed under a light microscope (Leica Microsystems, Germany) at 20x magnification. The pancreatic lesions were scored in five random fields per section in a blinded manner using a semiquantitative grading system (26). The samples were graded according to the percentage area of the lesions as follows: 0 = absent; 1 = rare; 2 = minimal, involving <10% of the total parenchyma; 3 = moderate, involving <50% of the total parenchyma; and 4 = major, involving >50% of the total parenchyma. The following pathological features were also used for evaluation: glandular atrophy (0 = absent; 1 = minimal; 2 = moderate; 3 = severe), fibrosis (0 = absent; 2 = confined to abnormal areas; 4 = diffuse), and presence of pseudotubular complexes (0 = absent; 1 = minimal; 2 = moderate; 3 = major). The total score for each pancreatic section was calculated. In addition, the tissue sections were stained with Sirius Red staining solution (Chondrex Inc., WA, USA) to assess collagen deposition. The stained collagen fibers were viewed under a light microscope at 20x magnification, and the Sirius red-positive area was quantified in five microscopic fields per section using ImageJ software.

### 2.6 Alcian blue-periodic acid-schiff staining

Colonic tissues were harvested immediately after euthanizing the mice, and immersed overnight in 4% paraformaldehyde. The fixed tissue specimens were dehydrated using graded ethanol, clarified in xylene, embedded in paraffin, and then cut into 5-μm-thick serial sections with a Leica rotary microtome (Leica Biosystems, Germany). Following deparaffinization and rehydration, the sections were stained with 1% Alcian blue in 3% glacial acetic acid (pH 2.5) for 30 min at room temperature to label acidic mucins. The slides were placed under running tap water for 2 min to wash off the excess stain, rinsed with distilled water, and immersed in 1% periodic acid solution for 5 min. After rinsing with distilled water, the sections were incubated with Schiff’s reagent for 10 min at room temperature to label neutral mucins. Excess dye was washed off with running tap water for 5 min to allow the color to develop. The sections were then counterstained with hematoxylin for 1 minute, washed under running tap water, dehydrated in graded ethanol, cleared in xylene, and mounted with neutral resin.

### 2.7 Immunohistochemistry

Pancreatic tissues were harvested, fixed in 4% paraformaldehyde overnight, dehydrated with graded ethanol, cleared in xylene, embedded in paraffin, and serially sectioned at 5 μm-thick sections with a Leica rotary microtome (Leica Biosystems, Germany) for subsequent histological staining and analysis. The tissue sections were immersed in hot Tris-EDTA buffer (pH=9) in a pressure cooker for 2 min for unmasking the antigens, and then covered with 3% hydrogen peroxide to block the endogenous peroxidases. To inhibit non-specific binding, the sections were incubated with 10% goat serum for 30 min at 37 °C. The sections were then incubated overnight with anti-IL-1β (Proteintech, 16806-1-AP, 1:100), anti-IL-6 (Proteintech, 21865-1-AP, 1:100), anti-TNF-α (Proteintech, 60291-1-Ig, 1:100), anti-MPO (Proteintech, 22225-1-AP, 1:100), anti-α-SMA (Proteintech, 67735-1-Ig, 1:100), and anti-collagen I (Proteintech, 66753-1-Ig, 1:100) antibodies at 4 °C. After washing once, the sections were incubated with HRP-conjugated secondary antibody for 30 min at 37 °C, followed by diaminobenzidine (DAB) for 5–10 min to initiate chromogenic reaction. Images were acquired with an optical microscope, and the positively stained regions were quantified using ImageJ software.

### 2.8 Immunofluorescence

Pancreatic and colonic paraffin sections were deparaffinized and rehydrated, followed by antigen retrieval in citrate buffer (Proteintech, Wuhan, China, Cat. No. PR10005) using microwave heating at medium–high power for 30 min. The sections were blocked with 5% BSA (Beyotime, Jiangsu, China, Cat. No. ST2254) for 1 h. Sequential multiplex staining was performed according to the manufacturer’s protocol of the Tyramide Signal Amplification (TSA) Fluorescence Double Staining Kit (G1226, Servicebio, China). Briefly, each target antigen was labeled sequentially: primary antibody incubation at 4 °C overnight, incubation with HRP-conjugated goat anti-rabbit IgG (Servicebio, GB23303, 1:300), fluorophore-tyramide deposition, and antibody stripping before the next labeling cycle. The primary antibodies used were anti-CD206 (Proteintech, 83485-1-RR, 1:500), anti-iNOS (Proteintech, 18985-1-AP, 1:100–1:500), anti-F4/80 (Abcam, ab111101, 1:100), anti-claudin 1 (Proteintech, 13050-1-AP, 1:1000), anti-occludin (Proteintech, 80545-1-RR, 1:500), and anti-ZO-1 (Proteintech, 82870-1-PBS, 1:800). After all sequential labeling procedures, nuclei were counterstained with 4′,6-diamidino-2-phenylindole (DAPI). Images were acquired with a fluorescence microscope (Leica, Wetzlar, Germany, DM2500), and analyzed with Pannoramic SCAN II (3D HISTECH) and LAS X software (Leica, Wetzlar, Germany).

### 2.9 Western blotting

The protein fraction of pancreatic and colonic tissues was extracted using RIPA lysis buffer, and quantified using a BCA assay kit (KeyGEN BioTECH, Nanjing, China; Cat. No. KGP902). Equivalent protein amounts per sample were separated by SDS-PAGE (8%-12% PA gel) and transferred onto PVDF membranes. After blocking with 5% non-fat milk at room temperature for 1 h, the membranes were incubated overnight with anti-P65 (CST, 8242, 1:1000), anti-pP65 (CST, 3033, 1:1000), anti-p-AMPKα (Thr172) (CST, 2535, 1:1000), anti-AMPKα (CST, 5831, 1:1000), anti-TLR4 (CST, 38519, 1:1000), anti-PPARα (CST, 9800, 1:1000), anti-PGC-1α (CST, 2178, 1:1000), anti-p-ACC (Ser79) (CST, 11818, 1:1000), and anti-ACC (CST, 3676, 1:1000) antibodies at 4 °C. After washing with PBST, the membranes were incubated with HRP-conjugated goat anti-rabbit IgG (Proteintech, SA00001-2, 1:5000) for 1 h at room temperature, and washed again with PBST. The protein bands were visualized using the Odyssey CLx imaging system (LI-COR Biosciences, Lincoln, USA), and band densities were quantified using ImageJ software, with β-actin as the loading control.

### 2.10 Quantification of *A. muciniphila* colonization

Total genomic DNA was extracted from fecal samples using a Stool DNA Kit (Omega Bio-tek, Norcross, GA, USA; Cat. No. D4015) according to the manufacturer’s instructions. The abundance of *A. muciniphila* was quantified by qPCR using species-specific primers and normalized to total bacterial 16S rRNA gene levels. qPCR was performed using ChamQ Universal SYBR qPCR Master Mix (Vazyme Biotech Co., Ltd., Nanjing, China; Cat. No. Q711-02), and relative abundance was calculated using the 2^ -ΔΔCt method. Primers were synthesized by Sangon Biotech, and their sequences are listed in **Table 1**.

**Table 1:** Primer sequences used for bacterial quantification.

Target	Forward primer (5’-3’)	Reverse primer (5’-3’)
16S rRNA(total bacteria)	ACTCCTACGGGAGGCAGCAG	ATTACCGCGGCTGCTGG
*A. muciniphila*	AGAGTTTGATCCTGGCTCAG	GGTTACCTTGTTACGACTT

### 2.11 Quantitative real-time PCR

Total RNA was extracted from pancreatic tissue using an RNA Extraction Kit (Vazyme Biotech Co., Ltd., Nanjing, China; Cat. No. RMA3302) according to the manufacturer’s protocol. Complementary DNA (cDNA) was synthesized by reverse transcription using the PrimeScript™ FAST RT reagent Kit with gDNA Eraser (Vazyme Biotech Co., Ltd., Nanjing, China; Cat. No. R323-01). RT-qPCR was performed using ChamQ Universal SYBR qPCR Master Mix (Vazyme Biotech Co., Ltd., Nanjing, China; Cat. No. Q711-02). The amplification program consisted of an initial denaturation at 95 °C for 3 min, followed by 40 cycles of 95 °C for 10 s and 60 °C for 30 s. Melting curve analysis was performed to verify amplification specificity. The relative expression level of each mRNA was normalized to the reference gene β-actin and calculated using the 2^-ΔΔCt method. Primer sequences (synthesized by Sangon Biotech) are shown in **Table 2**.

**Table 2:** The primer sequences chosen in the RT-qPCR.

Gene name	Forward primer (5’-3’)	Reverse primer (5’-3’)
IL-1β	TCGCAGCAGCACATCAACAAGAG	AGGTCCACGGGAAAGACACAGG
IL-6	CTTCTTGGGACTGATGCTGGTGAC	AGGTCTGTTGGGAGTGGTATCCTC
TNF-α	CTGAACTTCGGGGTGATCGG	AGGGTCTGGGCCATAGAACT
IL-10	TCCCTGGGTGAGAAGCTGAAGAC	CACCTGCTCCACTGCCTTGC
Col1a1	GGATAG GGACTTGTGTGA	GCTGGA AGAGTGAAGAGG
α-SMA	GGACGTACAACTGGTATTGTGC	TCGGCAGTAGTCACGAAGGA
β-actin	GTGCTATGTTGCTCTAGACTTCG	ATGCCACAGGATTCCATACC

### 2.12 Gut microbiota analysis

To profile the structure of the intestinal microbial community, 16S rRNA gene amplicon sequencing was implemented. Library preparation and high-throughput sequencing services were provided by Novogene Biotech Co., Ltd. (Beijing, China). In brief, fresh colon luminal contents were carefully collected from individual mice and preserved in sterile tubes at −80 °C. Total genomic DNA was isolated from specimens via the hexadecyltrimethylammonium bromide (CTAB) extraction method. After quality and concentration assessment, recovered DNA was normalized to 1 ng/μL. The V3–V4 hypervariable region of bacterial 16S rRNA genes was amplified by PCR with primer pairs 341F (5′-CCTAYGGGRBGCASCAG-3’) and 806R (5′-GGACTACNNGGGTATCTAAT-3′). Combined PCR amplicons were purified employing the Qiagen Gel Extraction Kit (Qiagen, Germany). Sequencing libraries were generated using the TruSeq DNA PCR-Free Sample Preparation Kit (Illumina, USA). Library quality was verified with a Qubit@ 2.0 Fluorometer (Thermo Scientific) and the Agilent Bioanalyzer 2100 system. The constructed libraries were subsequently sequenced on the Illumina NovaSeq platform. UPARSE software (version 7.0.1001) was adopted to cluster sequences sharing ≥97% identity into operational taxonomic units (OTUs). After normalization of OTU abundance matrices, a series of downstream diversity analyses were undertaken to compare the diversity, taxonomic composition, community complexity, and relative abundance of gut microbiota among experimental groups. Raw 16S rRNA gene sequencing reads have been deposited in the NCBI Sequence Read Archive (SRA) under BioProject accession number PRJNA1506688.

### 2.13 Targeted metabolomics for SCFA measurement

Mouse fecal samples and A. muciniphila culture supernatants were collected for targeted SCFA metabolomic analysis performed by Novogene Biotech Co., Ltd. (Beijing, China). Fecal samples were diluted 1:10 (w/v) and mixed with an equal volume of 50% aqueous acetonitrile for protein precipitation. After thorough mixing, samples were centrifuged (10,000 g, 10 min, 4 °C). The resulting supernatants of fecal extracts and bacterial culture supernatants were analyzed for SCFAs using liquid chromatography–tandem mass spectrometry (LC–MS/MS). Fecal SCFA concentrations were normalized to fecal wet weight and expressed as ng/g fecal weight, while SCFA concentrations in bacterial culture supernatants were presented as ng/mL.

### 2.14 Statistical analysis

Data are presented as means ± standard error of the mean (SEM). Statistical analyses were performed using GraphPad Prism 8.0, SPSS 21.0, and R 3.6.0. Unpaired Student’s t-test was used for two-group comparisons. One-way analysis of variance (ANOVA) with Tukey’s multiple comparisons test was adopted for multiple-group comparisons. Spearman’s rank correlation analysis was used to assess the associations between variables.A P value < 0.05 was considered statistically significant.

## 3 Results

### 3.1 SCO-792 ameliorated HFD-aggravated pancreatic injury and fibrosis in mice

**Figure 1:**
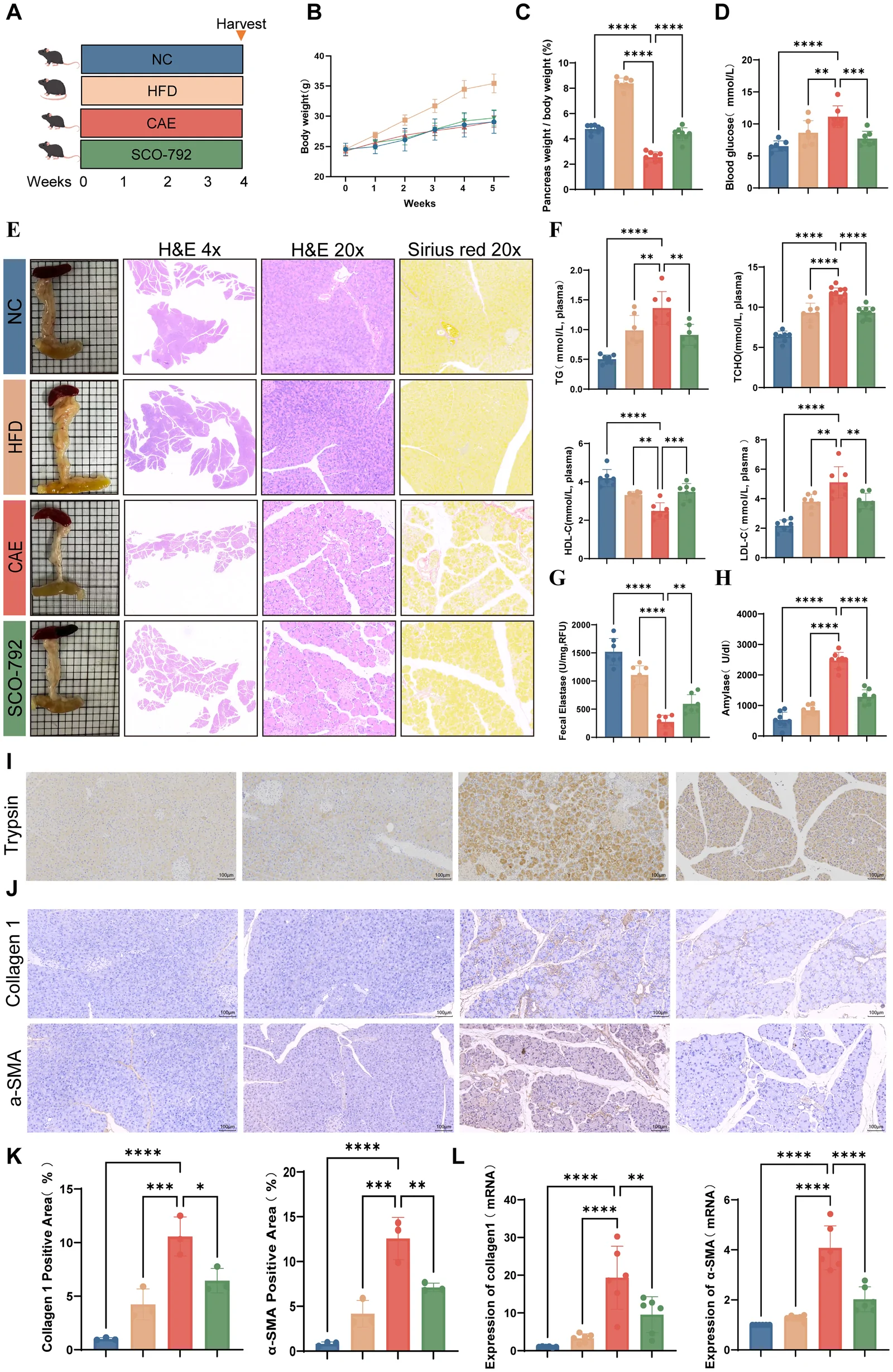
Effects of SCO-792 on pancreatic injury and fibrosis in mice with HFD-aggravated CP. **(A)** Schematic diagram of CP modeling and intervention protocol. **(B)** Body-weight changes of mice across groups over the 5-week experimental period. **(C)** Pancreas-to-body weight ratio of mice in the indicated groups. **(D)** FBG levels in mice in the indicated groups. **(E)** Gross morphological views of mouse pancreas (left panel), H&E-stained pancreatic sections (middle panel, 4× and 20× magnification), and Sirius-red-stained pancreatic sections (right panel, 20× magnification) from indicated groups. Scale bar = 100 μm. **(F)** Serum levels of TG, TC, HDL-C, LDL-C in the indicated groups. **(G)** Fecal elastase-1 activity across experimental groups. **(H)** Plasma amylase activity in mice from indicated groups. **(I)** Selected IHC micrographs of trypsin in the pancreatic tissues of the indicated groups. Scale bar = 100 μm. **(J)** IHC micrographs for Collagen I and α‑SMA in pancreatic tissues from indicated groups. Scale bar = 100 μm. **(K)** Percentage positive area of collagen 1 and α-SMA IHC staining in the pancreatic tissues of the indicated groups. **(L)** Relative expression levels of collagen 1 and α-SMA mRNAs in the pancreatic tissues of the indicated groups. Groups: NC, normal control group; HFD, high-fat diet control group; CAE, caerulein-induced chronic pancreatitis model group with HFD feeding; SCO-792, SCO-792 intervention group. Data are presented as mean ± SD, n = 6–8 mice per group. ns indicates no significant difference; *P < 0.05, **P < 0.01, ***P < 0.001, ****P < 0.0001.

The therapeutic effects of SCO-792 were evaluated in mice with HFD- and CAE-induced CP. The experimental workflow and grouping are shown in **Figure 1A**. HFD feeding led to a progressive, age-dependent increase in the body weight of mice compared to that in the NC group. On the other hand, mice in the CAE group showed a marked reduction in the pancreas-to-body weight ratio, along with elevated fasting blood glucose (FBG) levels (**Figures 1B–D**). Histological assessment of the pancreatic tissues from the different groups confirmed these pathological changes (**Figure 1E**). The NC group displayed intact pancreatic architecture, with evenly arranged acini and no obvious signs of inflammatory infiltration or fibrosis. While HFD feeding alone did not induce any significant pathological alterations, the mice subjected to both HFD and CAE showed the typical pathological features of CP, including pronounced acinar atrophy, inflammatory cell infiltration, and interstitial fibrosis, along with elevated pathological score and increased collagen deposition. SCO-792 intervention significantly attenuated acinar injury, inflammatory infiltration, and collagen deposition, and reduced the pathological scores in CAE mice (**Figure 1E**). SCO-792 intervention partially reversed these changes in CAE mice, as indicated by reduced blood glucose levels and improved pancreatic weight ratio. Furthermore, the CAE mice also exhibited an aberrant serum lipid profile, characterized by significantly higher levels of TG, TC and LDL-C, and reduced HDL-C levels compared to the NC mice (**Figure 1F**). Fecal elastase-1 activity was markedly decreased in the CAE group relative to the NC group (**Figure 1G**). In addition, SCO-792 treatment restored the plasma lipid profile and pancreatic enzyme activities in CAE mice(**Figure 1H**; **Supplementary Figure 1**). Consistent with the histological findings, the positively stained areas of trypsin, collagen 1, and α-SMA in the pancreatic tissues were more extensive in the CAE group compared to that in the NC group (**Figures 1I, J**), indicating significant pancreatic fibrosis. Likewise, SCO-792 treatment substantially reduced the positive expression areas of these fibrotic markers (**Figure 1K**), and downregulated fibrosis-related genes (**Figure 1L**) in the pancreatic tissues of CAE mice, suggesting that SCO-792 can inhibit the activation of pancreatic stellate cells. Taken together, SCO-792 alleviated pancreatic exocrine dysfunction, restored lipid metabolism, inhibited pancreatic stellate cell activation, and attenuated pancreatic fibrosis in mice with HFD-aggravated CP.

### 3.2 SCO-792 regulated pancreatic inflammation and macrophage polarization in mice with HFD-aggravated CP

**Figure 2:**
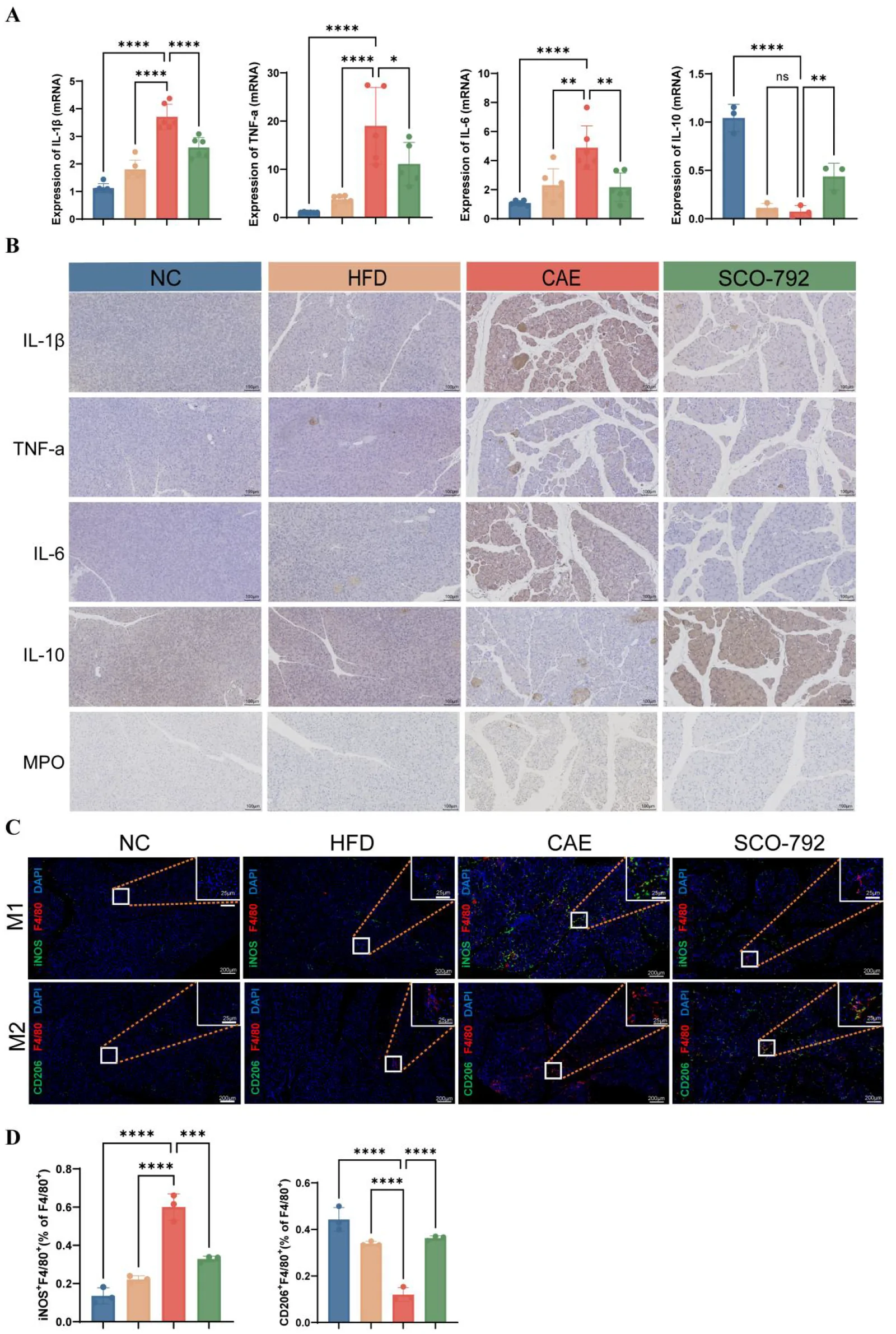
SCO-792 regulated pancreatic inflammation and macrophage polarization in mice with HFD-aggravated CP. **(A)** Relative expression levels of *IL-1β*, *TNF-α*, *IL-6*, and *IL-10* mRNAs in the pancreatic tissues of the indicated groups. **(B)** Selected IHC micrographs of IL-1β, TNF-α, IL-6, IL-10, and MPO in the pancreatic tissues of the indicated groups. Scale bar = 100 μm. **(C)** Representative IF co-staining images of F4/80 (red) with CD206 (green) and iNOS (green) in the pancreatic tissues of the indicated groups. Nuclei were counterstained with DAPI (blue). Scale bar = 200 μm. **(D)** Quantification of the percentage of iNOS^+^F4/80^+^ M1-like macrophages and CD206^+^F4/80^+^ M2-like macrophages among total F4/80^+^ macrophages. Groups: NC, normal control group; HFD, high-fat diet control group; CAE,caerulein-induced chronic pancreatitis model group with HFD feeding; SCO-792, SCO-792 intervention group. Data are presented as mean ± SD, n = 6–8 mice per group. ns indicates no significant difference; *P < 0.05, **P < 0.01, ***P < 0.001, ****P < 0.0001.

To investigate the potential mechanism underlying the therapeutic effects of SCO-792, we next evaluated pancreatic inflammation and macrophage polarization in the different groups. As shown in **Figure 2A**, the transcripts of pro-inflammatory cytokines, including IL-1β, TNF-α, and IL-6, were significantly upregulated, while that of the anti-inflammatory IL-10 was downregulated in the pancreatic tissues of CAE mice relative to the NC mice. SCO-792 intervention significantly downregulated IL-1β, TNF-α, and IL-6 mRNAs, and partially restored IL-10 mRNA expression. IHC staining validated the changes in cytokine levels; compared to the NC group, the CAE group showed markedly enhanced staining for the pro-inflammatory cytokines in the pancreatic tissues, along with significantly diminished IL-10 expression (**Figure 2B**). Additionally, the neutrophil marker MPO was substantially increased in the pancreas of the CAE mice, which was consistent with inflammatory infiltration (**Figure 2B**). SCO-792 intervention reduced the expression of these pro-inflammatory cytokines and MPO, and restored IL-10 expression. Furthermore, IF staining of the pancreatic tissues of CAE mice revealed significant co-localization of F4/80, the pan-macrophage marker, with the M1 macrophage-specific iNOS. On the other hand, the co-localization signal of the M2 macrophage marker CD206 with F4/80 was markedly diminished (**Figures 2C, D**). SCO-792 intervention reduced the infiltration of the pro-inflammatory M1 macrophages while increasing the abundance of M2 macrophages, indicating that SCO-792 can polarize pancreatic macrophages towards the anti-inflammatory phenotype. Taken together, SCO-792 alleviated the local pancreatic inflammatory microenvironment in mice with HFD-aggravated CP by suppressing the release of pro-inflammatory cytokines, inhibiting neutrophil infiltration, and promoting M1 to M2 polarization of resident macrophages.

### 3.3 SCO-792 restored intestinal barrier function in mice with HFD-aggravated CP

**Figure 3:**
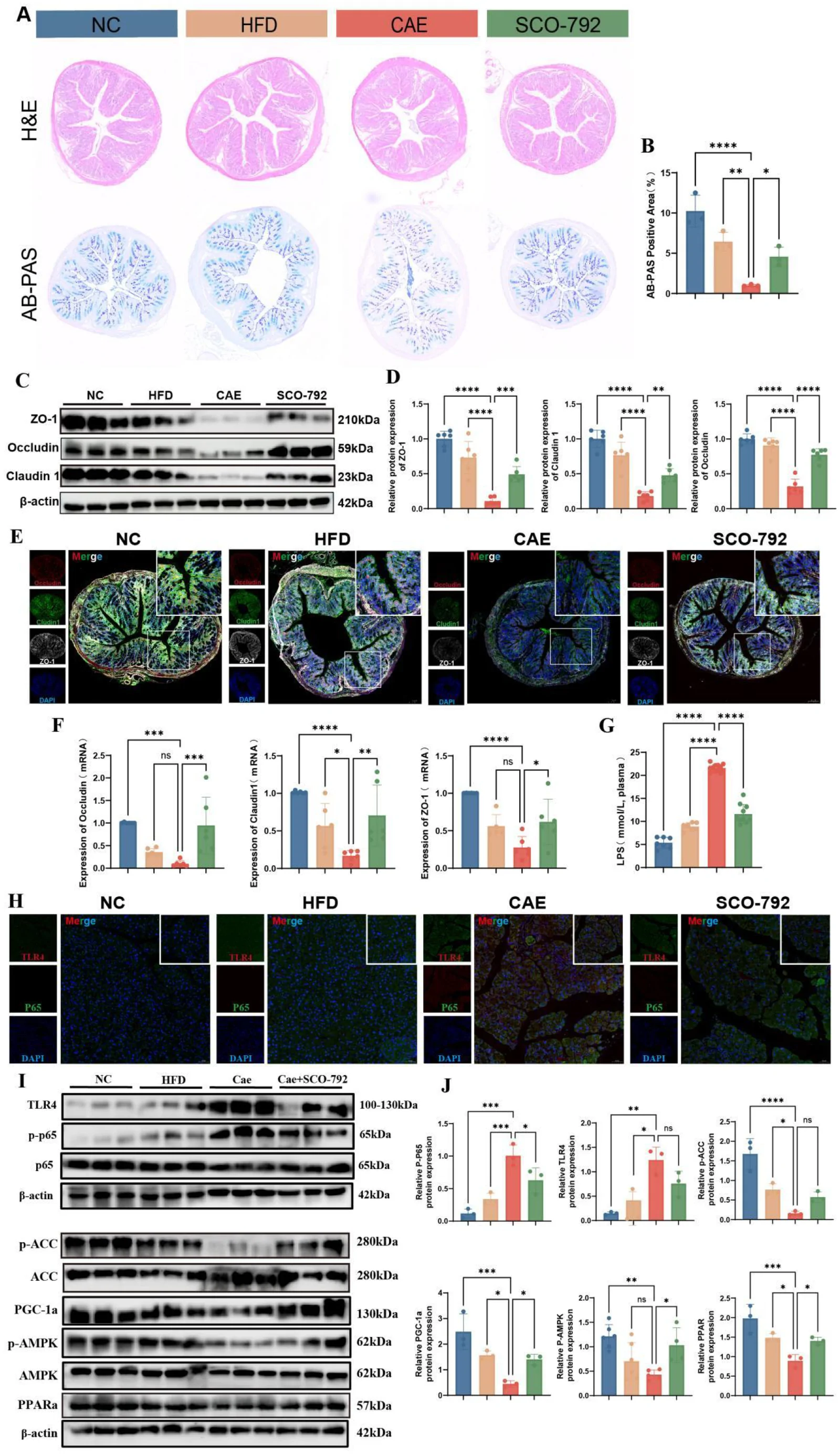
SCO-792 restored intestinal barrier function in mice with HFD-aggravated CP. **(A)** H&E staining (upper panel) and AB-PAS staining (lower panel) of colonic tissues from the indicated groups. Scale bar = 100 µm. Scale bar = 100 µm. **(B)** Percentage of AB-PAS positive staining area in the colonic tissues of mice in the indicated groups. **(C)** Immunoblot showing expression of ZO-1, Occludin, and Claudin-1 in the colonic tissues of the indicated groups. **(D)** Quantitative densitometric analysis of **(C)**, with β-actin as the loading control. **(E)** Representative IF staining images of Occludin (red), Claudin1 (green), and ZO-1 (white) in colonic tissues of the indicated groups. Nuclei were counterstained with DAPI (blue). Scale bar = 100 µm. **(F)** Relative expression levels of occludin, claudin-1, and ZO-1 mRNAs in the colonic tissues of the indicated groups. **(G)** Plasma LPS concentrations in mice from each group. **(H)** IF staining for TLR4 (red) and p65 (green) in colonic tissues. Nuclei were counterstained with DAPI (blue). Scale bar = 50 µm. **(I)** Immunoblot showing expression of TLR4/NF-κB pathway and AMPK/PPARα pathway proteins in the colonic tissues of the indicated groups. **(J)** Quantitative densitometric analysis of **(I)**, with β-actin as the loading control. Groups: NC, normal control group; HFD, high-fat diet control group; CAE, caerulein-induced chronic pancreatitis model group with HFD feeding; SCO-792, SCO-792 intervention group. Data are presented as mean ± SD, n = 6–8 mice per group. ns indicates no significant difference; *P < 0.05, **P < 0.01, ***P < 0.001, ****P < 0.0001.

To assess the impact of SCO-792 on intestinal barrier integrity, we examined colonic tissue morphology, tight junction (TJ) protein expression, and changes in related signaling pathways. The colonic tissues of CAE mice showed disrupted mucosal structure and a marked reduction in goblet cells compared to that of NC mice. SCO-792 intervention significantly ameliorated the colonic mucosal injury and increased goblet cell count (**Figures 3A, B**). Consistent with the histological findings, the TJ proteins ZO-1, occludin, and claudin-1 were significantly decreased in the colonic tissues of CAE mice, and showed irregular distribution (**Figures 3C–E**). SCO-792 intervention not only increased the expression of these TJ proteins but also restored their continuous distribution along the colonic epithelium. Furthermore, SCO-792 also increased the transcript levels of the TJ proteins, thus validating the above results (**Figure 3F**). The serum lipopolysaccharide (LPS) level was also markedly elevated in the CAE group compared to the NC group (**Figure 3G**), indicating increased endotoxin translocation due to intestinal barrier injury. SCO-792 intervention significantly reduced serum LPS levels, suggesting that it effectively decreased the entry of gut-derived endotoxin into the bloodstream. To elucidate the underlying mechanisms, we analyzed the changes in the expression of key proteins involved in the TLR4/NF-κB and AMPK/PPARα pathways in pancreatic tissues. IF staining revealed enhanced TLR4 fluorescence signals and prominent nuclear translocation of p65 in the pancreatic tissues of CAE mice (**Figure 3H**). Consistent with this, the TLR4 protein and the p-p65/p65 ratio were markedly elevated in the CAE group (**Figures 3I, J**). On the other hand, p-AMPK, p-ACC, PGC-1α, and PPARα levels were downregulated in the CAE group, which was indicative of AMPK/ACC pathway suppression. SCO-792 intervention inhibited the TLR4/NF-κB pathway and concurrently activated the AMPK/PPARα pathway. To summarize, SCO-792 ameliorates intestinal barrier injury in HFD-induced CP mice by upregulating intestinal TJ proteins to limit endotoxin translocation. In parallel, it suppresses the pro-inflammatory pancreatic TLR4/NF-κB pathway and activates the AMPK/PPARα signaling pathway, thereby alleviating pancreatic inflammation and injury.

### 3.4 SCO-792 remodeled the gut microbiome and enhanced microbial SCFAs production

**Figure 4:**
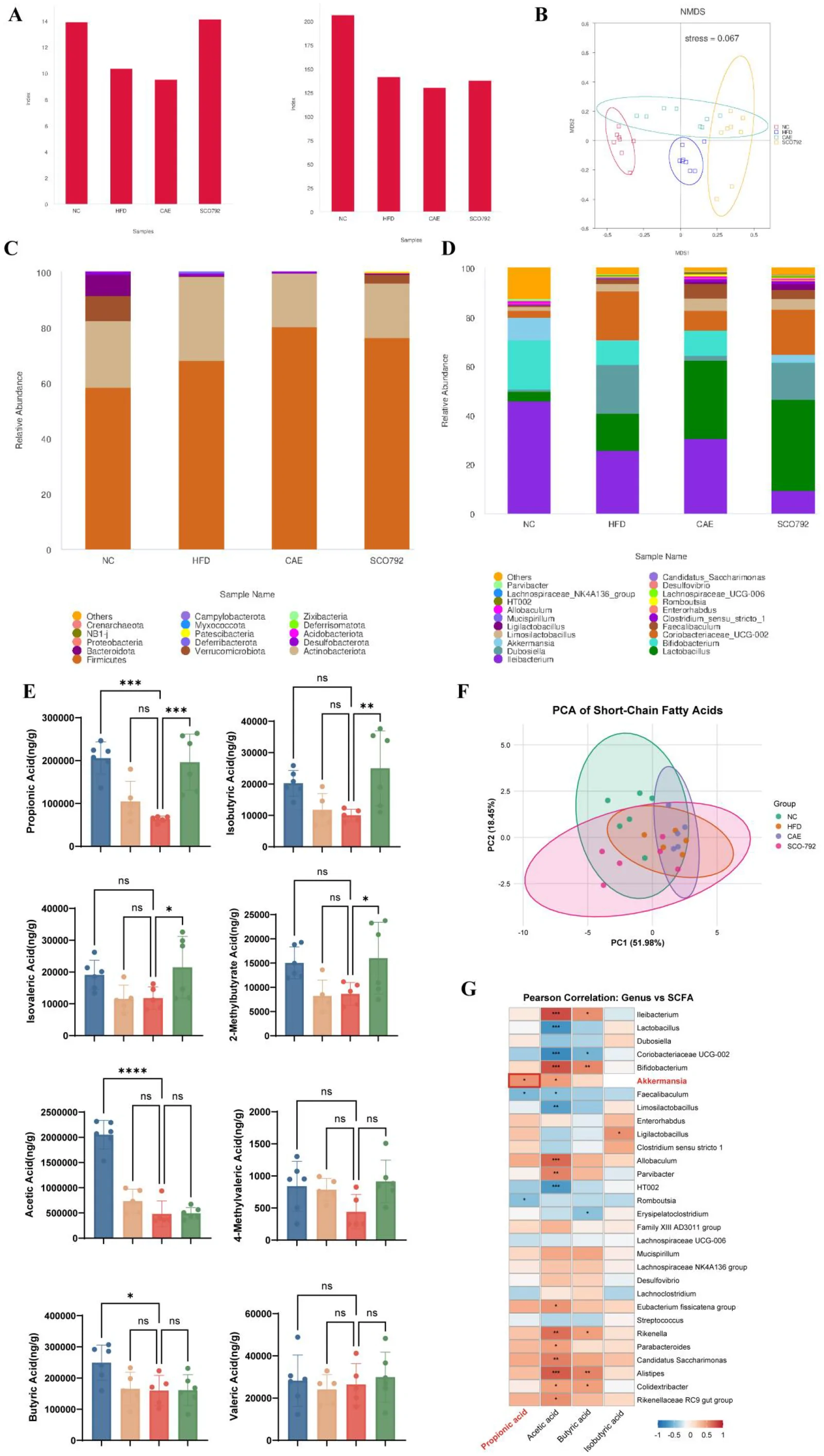
SCO-792 restored the composition of gut microbiota and SCFA production in mice with HFD-aggravated CP. **(A)** The α-diversity indices (Chao1, Shannon) of the intestinal microbiota in each group, showing microbial richness and diversity. **(B)** Principal coordinate analysis (PCoA) plot showing β-diversity and inter-group separation of the gut microbiota based on Bray-Curtis distances. **(C)** Bar plot showing relative abundance of different bacterial phyla in each group. **(D)** Heatmap showing relative abundance and distribution of different genera in each group. **(E)** Fecal SCFA levels in each group. **(F)** PCA plot of SCFA profiles, showing separation trends among groups. **(G)** Heatmap showing Spearman correlation coefficients between differentially abundant genera and SCFA levels. Groups: NC, normal control group; HFD, high-fat diet control group; CAE, caerulein-induced chronic pancreatitis model group with HFD feeding; SCO-792, SCO-792 intervention group. Data are presented as mean ± SD, n = 6–8 mice per group. ns indicates no significant difference; *P < 0.05, **P < 0.01, ***P < 0.001, ****P < 0.0001.

Given the potential role of the gut-pancreas axis in CP pathogenesis, we further analyzed the gut microbiota structure in the different groups by 16S rDNA sequencing. The results of α-diversity analysis revealed a significant reduction in gut microbial richness and diversity in the CAE group relative to the NC group, which was partially restored by SCO-792 intervention (**Figure 4A**). The β-diversity analysis was based on Bray-Curtis distances, and showed distinct microbial community structures of the different groups, with the CAE group deviating markedly from the NC group, and the SCO-792 intervention group shifting towards the NC group (**Figure 4B**). Phylum-level compositional analysis showed a significant reduction in the relative abundance of Bacteroidetes and an increased Firmicutes/Bacteroidetes ratio in the CAE group relative to the NC group. At the genus level, beneficial bacteria such as *Bifidobacterium* and *Lactobacillus* were markedly reduced, while potentially pathogenic bacteria were enriched in the CAE group. SCO-792 intervention reversed these changes, and significantly increased the relative abundance of *Akkermansia* (**Figures 4C, D**).

We also quantified fecal SCFAs, and found that the levels of acetic acid, butyric acid, isobutyric acid, propionic acid, and valeric acid were markedly lower in the CAE group compared to the NC group, and restored following SCO-792 intervention (**Figure 4E**). Principal component analysis (PCA) of the SCFA profiles revealed a clear separation between the CAE and NC groups, with the SCO-792 intervention group clustering closer to the NC group (**Figure 4F**). Furthermore, the abundance of *Lactobacillus*, *Bifidobacterium*, and other beneficial bacteria correlated positively with SCFA levels (**Figure 4G**), suggesting that SCO-792 may increase SCFA production by enriching these bacteria. Taken together, SCO-792 ameliorated gut dysbiosis, restored SCFA production, and reshaped the intestinal microecological balance in mice with HFD-induced CP.

### 3.5 Supplementation of *Akkermansia* and propionate protected against HFD-aggravated CP

SCO-792 intervention significantly increased the fecal abundance of *A. muciniphila* and propionate levels in HFD-fed mice with CP. *In vitro* anaerobic culture and targeted SCFA quantification confirmed that *A. muciniphila* produces propionate. Compared with blank medium supernatant, propionate concentration was markedly elevated in A. muciniphila culture supernatant (**Supplementary Figure 2**). Furthermore, molecular docking and molecular dynamics simulations revealed that propionate directly interacts with enteropeptidase(**Supplementary Figure 3**). Since *A. muciniphila* is a key propionate-producing species closely associated with host energy metabolism, we hypothesized that supplementation with this bacterium or its metabolite propionate may alleviate HFD-aggravated CP.

**Figure 5:**
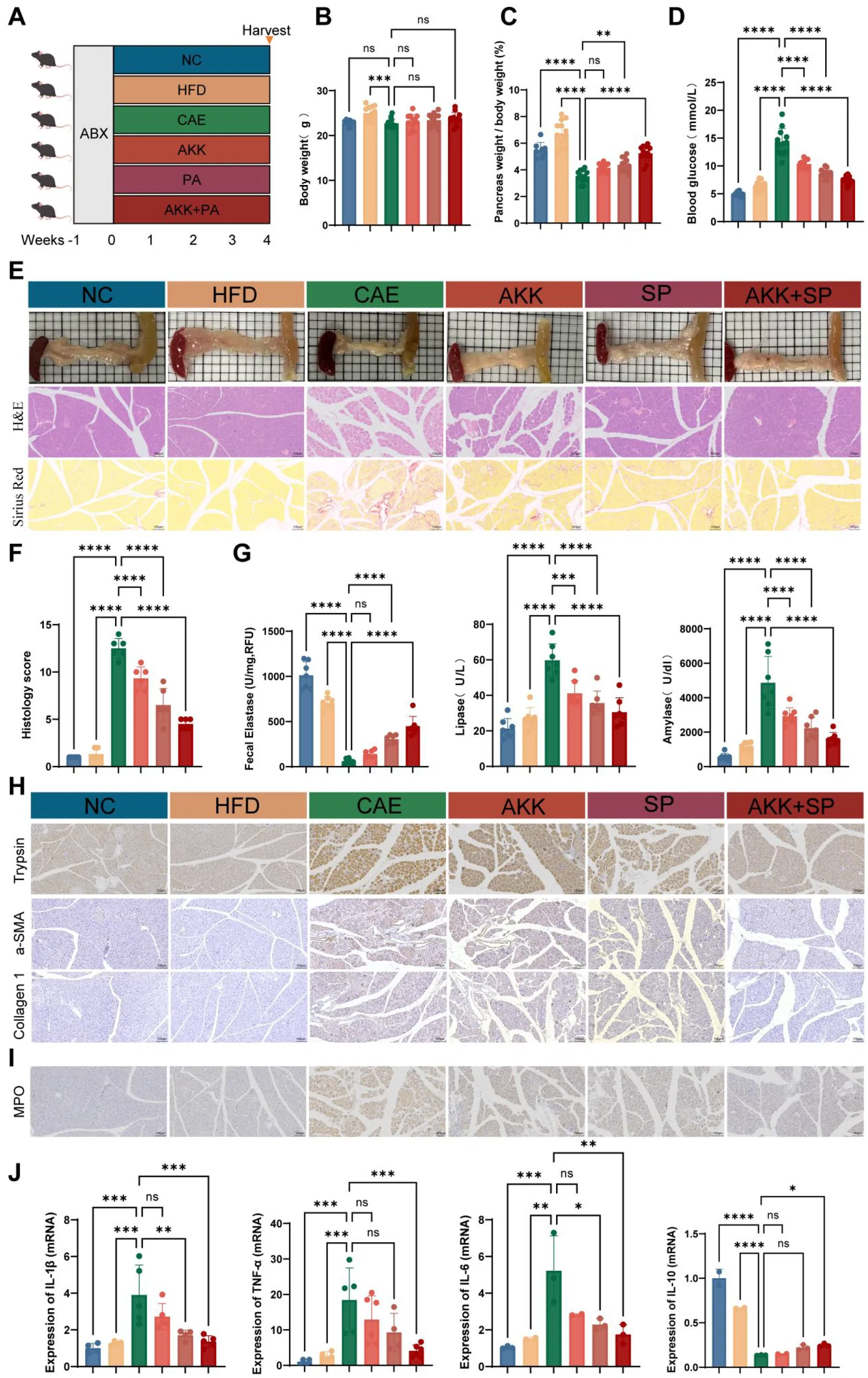
Supplementation of *Akkermansia* and propionate protected against HFD-aggravated CP. **(A)** Schematic diagram of CP modeling and intervention protocol with single or combined supplementation of AKK and PA. **(B–D)** Statistical analysis of pancreas-to-body weight ratio and FBG levels in different treatment groups. **(E)** Selected micrographs of pancreatic H&E staining and Sirius red staining in each group. Scale bar = 100 μm. **(F)** Quantitative pathological score analysis of pancreatic tissue. **(G)** Fecal elastase-1 levels, serum amylase and lipase activities in mice from the indicated groups. **(H, I)** Representative IHC staining images and quantitative positive area analysis of trypsin, collagen 1, α-SMA and MPO in pancreatic tissues. Scale bar = 100 μm. **(J)** Relative mRNA levels of inflammatory cytokines IL-1β, TNF-α, IL-6, IL-10 and neutrophil marker MPO in pancreatic tissues. Groups: CAE, caerulein-induced chronic pancreatitis model group with HFD feeding; AKK, Akkermansia muciniphila supplementation group; PA, propionate supplementation group; AKK + PA, combined intervention group. Data are presented as mean ± SD, n =6–8 mice per group. ns indicates no significant difference; *P < 0.05, **P < 0.01, ***P < 0.001, ****P < 0.0001.

To test this hypothesis, we established the CP model as illustrated in **Figure 5A**, and treated the mice with *A. muciniphila*, propionate, or their combination. After confirming successful colonization of *A. muciniphila* in the gut through qPCR analysis (**Supplementary Figure 4**).The general physiological parameters, histopathological indices, and fibro-inflammation markers were evaluated in the different treatment groups. Supplementation with *A. muciniphila* and/or propionate improved the pancreas-to-body weight ratio and serum FBG levels (**Figures 5B–D**). Furthermore, the different interventions also mitigated acinar atrophy, inflammatory infiltration, and interstitial fibrosis in the pancreatic tissues of CAE mice, and reduced collagen deposition (**Figures 5E, F**), while also restoring fecal elastase-1 activity and reducing serum amylase and lipase activities (**Figure 5G**). Notably, the combination of *A. muciniphila* and propionate achieved stronger ameliorative effects on the physiological and histopathological features of CP compared to either monotherapy. The extent of pancreatic fibrosis in the different groups was evaluated by measuring the *in situ* expression of trypsin, collagen 1, and α-SMA. All treatments reduced the expression of these fibrotic markers in the CAE group, and the combination group was most effective (**Figure 5H**). The effects of these treatments on pancreatic inflammation were assessed by measuring the levels of inflammatory cytokines and macrophage polarization. *A. muciniphila*, propionate, and their combination significantly reduced the levels of IL-1β, TNF-α, and IL-6, restored IL-10 expression, and downregulated the neutrophil marker MPO in the pancreatic tissues of CAE mice (**Figures 5I, J**). In summary, combined treatment with AKK and PA effectively ameliorated pancreatic exocrine dysfunction, alleviated lipid metabolism disorders, inhibited pancreatic stellate cell activation, reduced pro-inflammatory cytokine release and neutrophil infiltration, thereby delaying pancreatic fibrosis and improving the local inflammatory microenvironment. These results validate our hypothesis that AKK and propionate mimic the protective effects of SCO-792, and their combination exerts synergistic benefits.

### 3.6 Verification of the protective mechanism of SCO-792 via *A. muciniphila* and propionate supplementation

**Figure 6:**
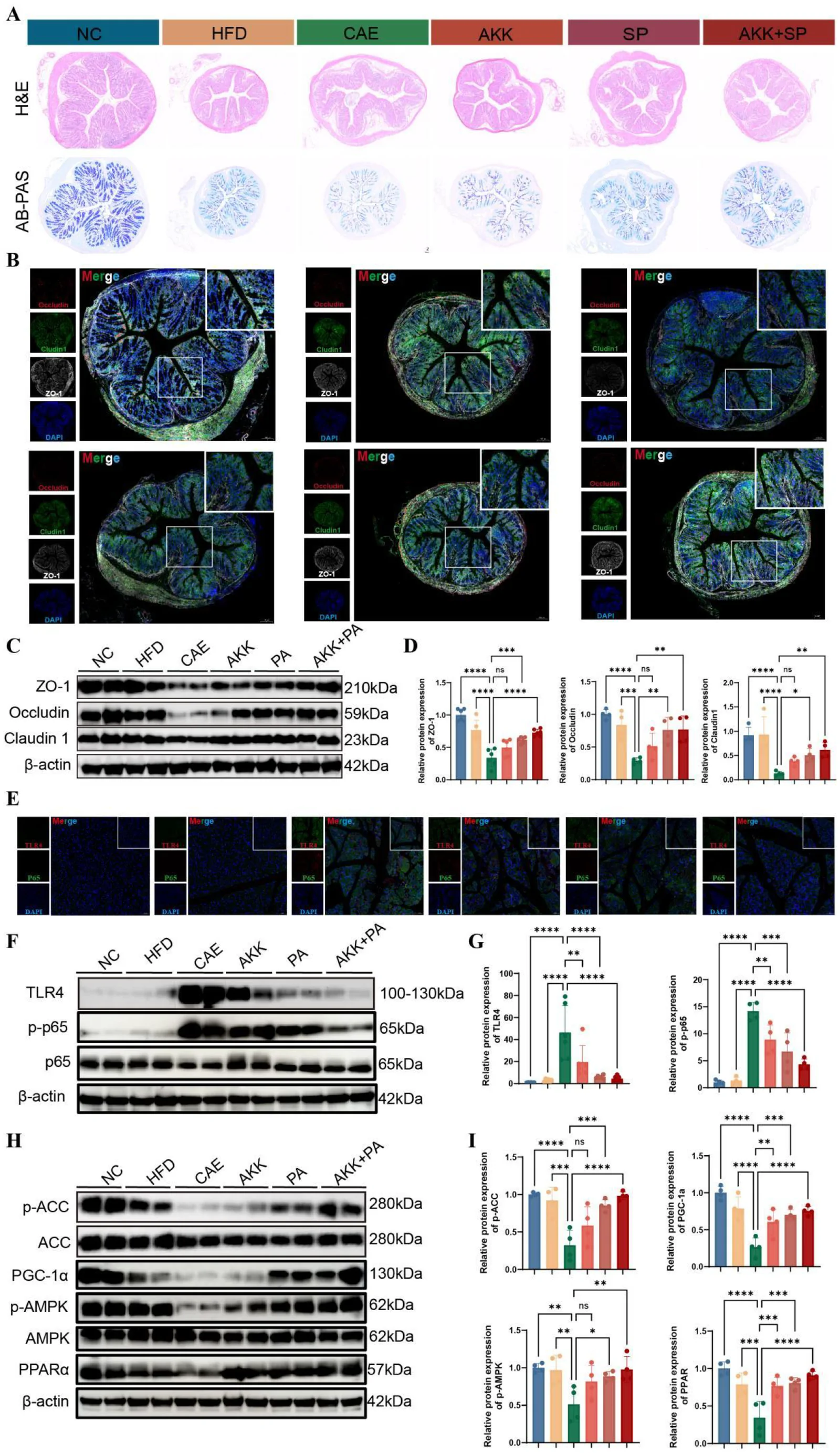
Effects of *A. muciniphila* and propionate on intestinal barrier function and related pathways in mice with HFD-aggravated CP. **(A)** Representative H&E staining (upper panel) and AB-PAS staining (lower panel) images of colonic tissues from each group, showing colonic mucosal morphology and goblet cell distribution. Scale bar = 100 μm. **(B)** Immunofluorescence labeling of occludin (red), claudin-1 (green), and ZO-1 (white) in colonic tissues.Nuclei were counterstained with DAPI (blue). Scale bar = 100 μm. **(C)** Immunoblot showing expression of TJ proteins in the colonic tissues of the indicated groups. **(D)** Quantitative densitometric analysis of **(C)**, with β-actin as the loading control. **(E)** Co-immunofluorescence staining for TLR4 (red) and NF-κB p65 (green) in colonic tissues. Nuclei were counterstained with DAPI (blue). Scale bar = 50 μm. **(F)** Immunoblot showing expression of TLR4, p65, and p-p65 in the pancreatic tissues of the indicated groups. **(G)** Quantitative densitometric analysis of **(E)**, with β-actin as the loading control. **(H)** Immunoblot showing expression of p-ACC, ACC, PGC-1α, AMPK, p-AMPK, and PPARα in the pancreatic tissues of the indicated groups. **(I)** Quantitative densitometric analysis of **(H)**, with β-actin as the loading control. Groups: CAE, caerulein-induced chronic pancreatitis model group with HFD feeding; AKK, Akkermansia muciniphila supplementation group; PA, propionate supplementation group; AKK + PA, combined intervention group. Data are presented as mean ± SD, n =6–8 mice per group. ns indicates no significant difference; *P < 0.05, **P < 0.01, ***P < 0.001, ****P < 0.0001.

To further validate the protective mechanism of SCO-792, we depleted the intestinal microflora of the CP-modeled mice using antibiotics, and administered *A. muciniphila* and propionate either alone or in combination. The experimental workflow is shown in **Figure 6A**. Supplementation of *A. muciniphila* and/or propionate significantly reduced the body weight and pancreatic index of CAE mice, and markedly improved serum amylase, lipase, and lipid profiles, with the combination treatment resulting in the most pronounced effects (**Figures 6B, C**). Furthermore, treatment with *A. muciniphila*, propionate, or their combination alleviated the structural disorganization, crypt atrophy, and goblet cell loss in the colonic tissues of CAE mice by varying degrees, with the combination group showing the most pronounced restoration of colonic mucosal architecture and goblet cell density (**Figure 6A**). Consistent with this, these microbiota-based interventions restored the expression levels and the continuous distribution of intestinal TJ proteins in the CAE mice (**Figures 6B–D**), and the most significant effects were observed in the combination group. To identify the mechanistic basis of improved intestinal barrier function, we analyzed the changes in the TLR4/NF-κB and AMPK/PPARα pathways. The colonic tissues of CAE mice showed increased TLR4 protein expression and p-p65/p65 ratio (**Figures 6E–G**), along with prominent nuclear translocation of NF-κB p65 (**Figure 6E**) compared to the NC group. Furthermore, the p-AMPK/AMPK ratio and PPARα expression levels were significantly downregulated in the CAE group compared to the NC group (**Figures 6H, I**). *A. muciniphila* and propionate intervention not only suppressed TLR4 expression and p65 phosphorylation, but also upregulated the AMPK pathway proteins, demonstrating superior efficacy compared to either monotherapy. To summarize, combined treatment with *A. muciniphila* and propionate restored intestinal barrier injury in mice with HFD-induced CP by upregulating TJ proteins and modulating the TLR4/NF-κB and AMPK/PPARα pathways, further confirming that SCO-792 exerts its protective effects by remodeling the gut microbiota and increasing propionate levels.

## 4 Discussion

CP is a progressive fibroinflammatory condition of the pancreas with few therapeutic options (1). High fat intake can significantly alter the gut microbiota structure, reduce microbial diversity and richness, and disrupt intestinal barrier integrity, resulting in the “leakage” of gut bacteria and their metabolites to extra-intestinal sites, where they trigger systemic low-grade inflammation (27, 28). This chronic inflammatory state exacerbates pancreatic tissue injury and fibrosis. Concurrently, CP can disturb lipid metabolism, establishing a vicious cycle in which metabolic disorder, dysbiosis, and pancreatic injury reinforce each other (29). The gut microbiota and its metabolites are increasingly being implicated in pancreatic diseases; this “gut-pancreas axis” offers a novel perspective for understanding the pathogenesis of CP and developing effective therapeutic strategies (30, 31). However, whether enteropeptidase inhibitors could ameliorate CP by modulating the gut-pancreas axis remained unclear. This study is the first to show that the enteropeptidase inhibitor SCO-792 can alleviate HFD-induced CP by regulating the network of gut microbiota, SCFAs, and the TLR4-AMPK-PPAR signaling pathway. Our findings not only establish a functional link between SCO-792 and the gut-pancreas axis, and reveal a novel mechanism of “remote” pancreatic protection via the modulation of intestinal microecology, but also support the application of enteropeptidase inhibitors for the treatment of CP.

As already mentioned, SCO-792 exerts its protective effects by remodeling the gut microbiota. In our study, SCO-792 intervention increased the α diversity of the intestinal microbiome in HFD-fed mice, and enriched beneficial bacteria like *Akkermansia* and other SCFAs-producing genera. *Akkermansia* is a mucin degrading commensal bacterium that colonizes the mucus layer, and its reduced abundance is linked to obesity, diabetes, and inflammatory bowel disease. In addition, fecal *Akkermansia* abundance is markedly reduced in patients with AP, and correlates with more severe systemic inflammation. Furthermore, the *Akkermansia*-derived protein Amuc_1409 has been shown to inhibit severe AP-induced pancreatic injury (32). In a model of carboxymethyl cellulose-aggravated AP, *Akkermansia* supplementation ameliorated intestinal barrier injury and decreased pancreatitis severity (33). Consistent with these reports, SCO-792 treatment restored *Akkermansia* abundance in mice with HFD-aggravated CP.

Concurrently, fecal SCFA measurements showed a marked elevation in propionate levels in the SCO-792 treated mice. Studies have documented the anti-inflammatory and immunomodulatory effects of propionate, and its ability to maintain mucosal homeostasis in various disease models (34). In a model of HFD-aggravated severe AP, exogenous supplementation of propionate or the propionate-producing *Lactobacillus* species alleviated pancreatitis severity; furthermore, the combination of the bacteria and the metabolite exhibited synergistic effects, which was linked to regulation of the gut-pancreas axis (35). Based on the positive correlation between propionate elevation and increased *Akkermansia* abundance in our study, we surmised that SCO-792 may increase propionate levels by enriching SCFA-producing bacteria such as *Akkermansia*. Furthermore, *Akkermansia* correlated negatively with the levels of proinflammatory cytokines, which supported a functional link among the gut microbiota, SCFAs, and inflammation. Altogether, these results suggest that SCO-792 exerts its protective effects through a sequence of microbiota remodeling, SCFA elevation, and inflammation suppression. Indeed, SCO-792-induced *Akkermansia* enrichment was accompanied by the amelioration of pancreatic inflammation and injury, without any notable adverse effects. Nevertheless, it should be noted that the effects of *Akkermansia* depend on the disease. For instance, a recent study showed that *Akkermansia* supplementation exacerbated intestinal injury and mortality in mice following acute radiation exposure, thus underscoring the need to evaluate its function in pancreatitis within specific pathological environments (36).

Pancreatic inflammation is also linked to the intestinal barrier integrity. HFD intake can lead to the downregulation or mislocalization of intestinal epithelial TJ proteins, including ZO-1, claudin-1, and occludin, resulting in increased intestinal permeability (37). This HFD-aggravated barrier disruption is closely associated with gut dysbiosis, exemplified by altered microbial composition, increased proliferation of Gram negative bacteria, impaired SCFA production, and lower microbial diversity, which further compromises barrier integrity (28). Furthermore, the “leaky gut” facilitates translocation of LPS from the intestinal lumen into the bloodstream. The circulating LPS then triggers production of reactive oxygen species in pancreatic acinar cells by activating TLR4, which in turn promotes the release of IL-6, TNF-α, and other inflammatory cytokines, thereby exacerbating pancreatic tissue injury (38). In severe AP, translocation of intestinal bacteria and LPS is a central event leading to enterogenic infection and high mortality. Patients with CP often exhibit gut dysbiosis and barrier dysfunction, and the microbial metabolites in their bloodstream continuously activate proinflammatory pathways within the pancreas via the gut-pancreas axis, eventually driving fibrosis and disease progression (39). In the present study, SCO-792 intervention significantly restored colonic expression of ZO-1, occludin, and claudin1, reduced goblet cell loss, and decreased serum LPS levels. In addition, the expression and co-localization of TLR4 and p-p65 in pancreatic tissues were markedly reduced in the SCO-792-treated mice. These results demonstrate that SCO-792 repairs the intestinal barrier, reduces LPS translocation, and subsequently inhibits excessive activation of the pancreatic TLR4/NF-κB pathway.

Macrophages are the key responder cells to TLR4 signaling. In our study, while the total number of F4/80 positive pancreatic macrophages remained unchanged following SCO-792 treatment, the proportion of iNOS+ M1 macrophages decreased significantly and that of CD206+ M2 macrophages increased, indicating a shift of the pancreatic microenvironment from a proinflammatory to an anti-inflammatory state. These findings are concordant with the established role of the LPS-TLR4 pathway in pancreatitis pathogenesis, and further support the notion that SCO-792 exerts its protective effects by blocking this pathogenic axis. Of note, LPS can also affect distal organs through the gut-brain axis, as exemplified by the disruption of the blood brain barrier and neuroinflammation, reflecting its systemic pathogenic potential. Therefore, the restorative effect of SCO-792 on the intestinal barrier may confer broader health benefits (40).

SCO-792 treatment also increased pancreatic p-AMPK levels and restored p-ACC expression, a downstream substrate of AMPK involved in regulating fatty acid synthesis. Concurrently, transcription factors involved in fatty acid oxidation (FAO), including PPARα and PGC-1α, were upregulated following SCO-792 treatment. In other words, the activated AMPK-PPARα-PGC-1α axis reduced *de novo* fatty acid synthesis by inhibiting ACC, and decreased lipid accumulation by promoting FAO, thereby restoring both systemic and pancreatic lipid metabolism. Furthermore, p-AMPK levels showed a significant negative correlation with serum TG and TC levels, and PPARα expression was negatively correlated with p-p65 levels, suggesting a close association between metabolic improvement and inflammation suppression. Activation of PPARα can further inhibit the NF-κB signaling pathway; while PPARα binds directly to the p65 subunit of NF-κB and prevents its nuclear translocation and transcriptional activity, PPARα indirectly inhibits NF-κB by inducing IκBα expression. This mechanism was corroborated by the negative correlation between PPARα expression levels and that of p-p65, TNF-α, and IL-6. Based on these results, we propose that TLR4 inhibition, AMPK activation, PPARα upregulation, and NF-κB suppression may form a coordinated regulatory network. In this network, AMPK activation may promote PPARα expression, whereas increased PPARα may inhibit NF-κB signaling and reduce inflammatory cytokine production, thereby attenuating inflammation-mediated suppression of AMPK activity. This regulatory interaction may contribute to the sustained protective effect of SCO-792.

To further verify that the protective effects of SCO-792 are mediated by the gut microbiota and SCFAs, we depleted the intestinal microflora of HFD-fed mice using antibiotics, and administered *Akkermansia* and/or propionate. Supplementation with either *Akkermansia* or propionate significantly ameliorated the pancreatitis phenotype, and their combination achieved greater efficacy. These results clearly demonstrate that SCO-792-induced gut microbiota remodeling and increased propionate production are key mediators of its protective effects, rather than being mere epiphenomena. Notably, propionate supplementation group not only restored intestinal barrier function but also activated the pancreatic AMPK pathway and upregulated PPARα expression, further supporting the existence of a “SCFAs-TLR4-AMPK-PPARα” signaling cascade.

This study has several limitations. First, as an enteropeptidase inhibitor, SCO-792 inhibits intestinal protein digestion and amino acid absorption, thereby disturbing systemic amino acid homeostasis and energy metabolism. Since such metabolic alterations may also contribute to pancreatic protection, it remains difficult to distinguish the direct protective effects of SCO-792 from indirect effects mediated by metabolic remodeling. Future studies using pair-feeding, amino acid supplementation, or intestine-specific enteropeptidase knockout will help clarify this issue. Second, although supplementation with or propionate alleviated chronic pancreatitis, these findings cannot rigorously confirm that they are necessary downstream mediators of SCO-792. SCO-792 treatment with or without microbiota depletion, or fecal microbiota transplantation using SCO-792-treated donors, would provide stronger causal evidence. Constrained by the initial study design, these experiments were not performed herein and will be explored in follow-up work. Third, we did not systematically verify the dose-dependent effects of propionate on tight junction proteins and the related downstream signaling pathways using *in vitro* cell or organoid models. Finally, our conclusions lack validation with clinical samples from patients with chronic pancreatitis; the therapeutic efficacy and gut-regulating effects of SCO-792 in humans remain to be confirmed by clinical trials.

To summarize, the enteropeptidase inhibitor SCO-792 alleviates HFD-aggravated CP by modulating the intestinal microecology and improving host lipid metabolism, and the two mechanisms act synergistically to form a positive feedback loop. Our findings support the clinical translation of SCO-792, and highlight the significance of the interplay between gut microbiota and host metabolism in CP pathogenesis. Furthermore, *Akkermansia* and propionate warrant functional validation and clinical exploration as potential probiotics or postbiotics for the management of CP.

## 5 Conclusion

**Figure 7:**
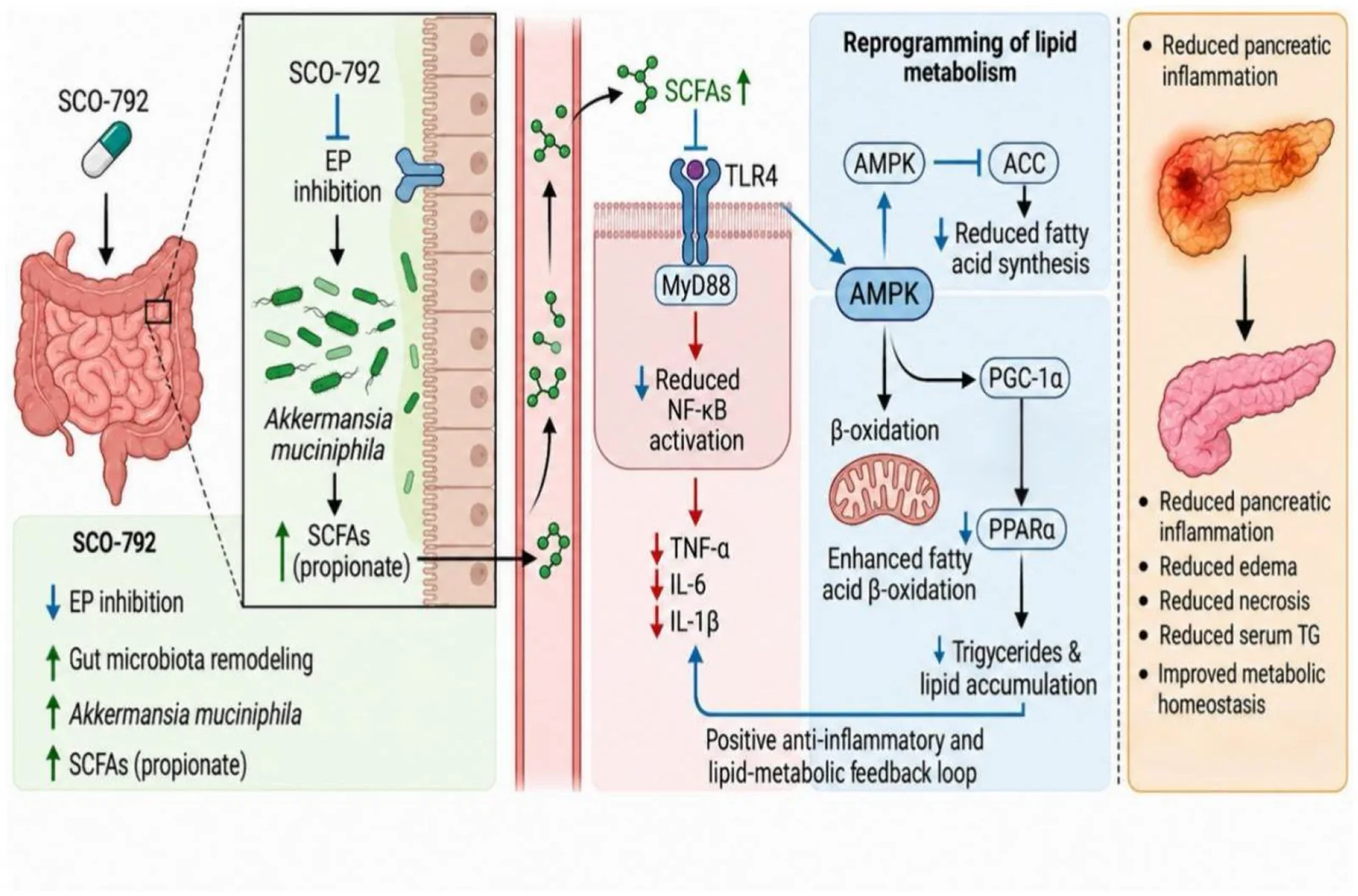
SCO-792 enriches propionate-producing *Akkermansia muciniphila* and modulates pancreatic inflammation and lipid metabolism through the gut-pancreas axis, ameliorating HFD-aggravated chronic pancreatitis.

This study demonstrates that the enteropeptidase inhibitor SCO‑792 protects against HFD‑aggravated chronic pancreatitis through the gut‑pancreas axis. By reshaping intestinal microecology and elevating propionate production, SCO‑792 preserves intestinal barrier integrity and blunts systemic endotoxin‑mediated pancreatic inflammation, while maintaining pancreatic lipid homeostasis via the AMPK‑PPAR signaling axis. Intestinal microbial metabolites serve as key mediators linking gut homeostasis to pancreatic metabolic‑inflammatory status. The therapeutic benefit of SCO‑792 arises from synergistic crosstalk between restored gut microecology and improved metabolic regulation, forming a positive feedback loop (**Figure 7**). Our results identify SCO‑792 as a promising pre‑clinical approach for metabolic‑associated chronic pancreatitis and support its clinical translation. Furthermore, Akkermansia and propionate warrant further functional validation and clinical exploration as potential probiotics or postbiotics for the management of CP.

## Data Availability

The data presented in the study are deposited in the NCBI Sequence Read Archive (SRA) repository, accession number PRJNA1506688.

## References

[1] Thierens N Verdonk RC Löhr JM van Santvoort HC Bouwense SA van Hooft JE . Chronic pancreatitis. Lancet (London England). (2025) 404:2605–18. doi: 10.1016/S0140-6736(24)02187-1 39647500

[2] Gupta A Chernyak V Chang KJ Bhosale PR Cash BD Hall KR . ACR appropriateness criteria® Chronic pancreatitis. J Am Coll Radiol JACR. (2026) 23:472–82. doi: 10.1016/j.jacr.2025.12.014 41493264

[3] Cohen SM Kent TS . Etiology, diagnosis, and modern management of chronic pancreatitis: A systematic review. JAMA Surg. (2023) 158:652–61. doi: 10.1001/jamasurg.2023.0367 37074693

[4] Ru N Xu XN Cao Y Zhu JH Hu LH Wu SY . The impacts of genetic and environmental factors on the progression of chronic pancreatitis. Clin Gastroenterol Hepatol Off Clin Pract J Am Gastroenterol Assoc. (2022) 20:e1378–87. doi: 10.1016/j.cgh.2021.08.033 34461303

[5] Gu X Huang Z Ying X Liu X Ruan K Hua S . Ferroptosis exacerbates hyperlipidemic acute pancreatitis by enhancing lipid peroxidation and modulating the immune microenvironment. Cell Death Discov. (2024) 10:242. doi: 10.1038/s41420-024-02007-1 38773098 PMC11109150

[6] Yang X Chen J Wang J Ma S Feng W Wu Z . Very-low-density lipoprotein receptor-enhanced lipid metabolism in pancreatic stellate cells promotes pancreatic fibrosis. Immunity. (2022) 55:1185–1199.e8. doi: 10.1016/j.immuni.2022.06.001 35738281

[7] Subramanian S Soran H Sikora Kessler A McClain MR Eckel RH Rosenson RS . Prevention and treatment of hypertriglyceridemia-mediated acute pancreatitis: A narrative review. Eur J Internal Med. (2025) 23:106648. doi: 10.1016/j.ejim.2025.106648 41444050

[8] Deng H Peng K Zhang L Lu J Mei W Shi X . Clinical outcomes in a multicenter cohort involving 919 patients with hypertriglyceridemia-associated acute pancreatitis. Am J Gastroenterol. (2025) 120(10):2405–14. doi: 10.14309/ajg.0000000000003319120 39817674

[9] Gukovskaya AS Lerch MM Mayerle J Sendler M Ji B Saluja AK . Trypsin in pancreatitis: The culprit, a mediator, or epiphenomenon? World J Gastroenterol. (2024) 30:4417–38. doi: 10.3748/wjg.v30.i41.4417 39534420 PMC11551668

[10] Zheng XL Kitamoto Y Sadler JE . Enteropeptidase, a type II transmembrane serine protease. Front Biosci (Elite Ed). (2009) 1:242–9. doi: 10.2741/E23 19482641

[11] Rajalingamgari P Khatua B Summers MJ Kostenko S Chang YH Elmallahy M . Prospective observational study and mechanistic evidence showing lipolysis of circulating triglycerides worsens hypertriglyceridemic acute pancreatitis. J Clin Invest. (2024) 135:e184785. doi: 10.1172/JCI184785 39509346 PMC11684810

[12] Chen H Wang Y Zippi M Fiorino S Hong W . Oxidative stress, DAMPs, and immune cells in acute pancreatitis: Molecular mechanisms and therapeutic prospects. Front Immunol. (2025) 16:1608618. doi: 10.3389/fimmu.2025.1608618 40909291 PMC12405227

[13] Mann NS Mann SK . Enterokinase. Proc Soc For Exp Biol Med Soc For Exp Biol Med (New York NY). (1994) 206:114–8. doi: 10.3181/00379727-206-43728 8208733

[14] Song Q Peng L Yang X Xu J Zhang D Tian X . Structural insight into the CUB2 domain's role in enteropeptidase-mediated trypsinogen activation. PNAS. (2026) 123:e2521394123. doi: 10.1073/pnas.2521394123 41838910 PMC13012022

[15] Yang X Ding Z Peng L Song Q Zhang D Cui F . Cryo-EM structures reveal the activation and substrate recognition mechanism of human enteropeptidase. Nat Commun. (2022) 13(1):6955. doi: 10.1038/s41467-022-34364-9 36376282 PMC9663175

[16] Ikeda Z Kakegawa K Kikuchi F Itono S Oki H Yashiro H . Design, synthesis, and biological evaluation of a novel series of 4-guanidinobenzoate derivatives as enteropeptidase inhibitors with low systemic exposure for the treatment of obesity. J Med Chem. (2022) 65:8456–77. doi: 10.1021/acs.jmedchem.2c00463 35686954 PMC9234964

[17] Rashid MY Noor A Patel V Henin S Cuello-Ramírez A Al Kaabi AS . Role of SCO-792, a novel enteropeptidase inhibitor, in the prevention of post-endoscopic retrograde cholangiopancreatography pancreatitis. Cureus. (2021) 13:e13724. doi: 10.7759/cureus.13724 33833935 PMC8018875

[18] Yashiro H Hamagami K Hiyoshi H Sugama J Tsuchimori K Yamaguchi F . SCO‐792, an enteropeptidase inhibitor, improves disease status of diabetes and obesity in mice. Diabetes Obes Metab. (2019) 21:2228–39. doi: 10.1111/dom.13799 31144422 PMC6771630

[19] Sugama J Moritoh Y Yashiro H Tsuchimori K Watanabe M Pharmacological Research . Enteropeptidase inhibition improves obesity by modulating gut microbiota composition and enterobacterial metabolites in diet-induced obese mice. Pharmacol Res. (2021) 163:105337. doi: 10.1016/j.phrs.2020.105337 33276106

[20] Katayama Y Sugama J Suzuki T Ishimura Y Kobayashi A Moritoh Y . Enteropeptidase inhibitor SCO-792 effectively prevents kidney function decline and fibrosis in a rat model of chronic kidney disease. Nephrol Dialysis Transplant Off Publ Eur Dialysis Transplant Assoc Eur Renal Assoc. (2021) 36:631–40. doi: 10.1093/ndt/gfaa349 33351150 PMC8008362

[21] Kagawa T Sugama J Nishizaki H Moritoh Y Watanabe M . An exploratory randomized trial of SCO-792, an enteropeptidase inhibitor, in patients with type 2 diabetes and albuminuria. Kidney Int Rep. (2022) 8:115–25. doi: 10.1016/j.ekir.2022.10.006 36644351 PMC9831944

[22] Hong J Fu Y Chen X Zhang Y Li X Li T . Gut microbiome changes associated with chronic pancreatitis and pancreatic cancer: A systematic review and meta-analysis. Int J Surg (London England). (2024) 110:5781–94. doi: 10.1097/JS9.0000000000001724 38847785 PMC11392207

[23] Zhu Y Liang X Zhi M Li L Zhang G Chen C . Succession of the multi-site microbiome along pancreatic ductal adenocarcinoma tumorigenesis. Front Immunol. (2024) 15:1487242. doi: 10.3389/fimmu.2024.1487242 39575247 PMC11580624

[24] Pan LL Ren ZN Yang J Li BB Huang YW Song DX . Gut microbiota controls the development of chronic pancreatitis: A critical role of short-chain fatty acids-producing Gram-positive bacteria. Acta Pharm Sin B. (2023) 13:4202–16. doi: 10.1016/j.apsb.2023.08.002 37799394 PMC10547962

[25] Machuca J Wirkus J Ead AS Cheon CY Mackenzie GG Oteiza PI . A dietary switch from a high fat to a low fat diet mitigates obesity-induced intestinal barrier dysfunction in mice: Implications for pancreatic carcinogenesis. Food Funct. (2025) 16:6160–73. doi: 10.1039/d5fo02372f 40662388

[26] Demols A Van Laethem JL Quertinmont E Degraef C Delhaye M Geerts A . Endogenous interleukin-10 modulates fibrosis and regeneration in experimental chronic pancreatitis. Am J Physiol Gastrointestinal Liver Physiol. (2002) 282:G1105–12. doi: 10.1152/ajpgi.00431.2001 12016137

[27] Jia W Wang H Feng T Liu X Liu Z Li Z . Structural characterization of polysaccharide from <i>Flammulina velutipes</i> and its impact on hyperlipidemia through modulation of hepatic cholesterol metabolism and gut microbiota. Foods (Basel Switzerland). (2025) 14:3452. doi: 10.3390/foods14193452 41097621 PMC12523756

[28] Zeng N Wu F Lu J Li X Lin S Zhou L . High-fat diet impairs gut barrier through intestinal microbiota-derived reactive oxygen species. Sci China Life Sci. (2024) 67:879–91. doi: 10.1007/s11427-022-2283-4 37202543

[29] Sui Y Zhang T Ou S Li G Liu L Lu T . Statin therapy associated Lactobacillus intestinalis attenuates pancreatic fibrosis through remodeling intestinal homeostasis. NPJ Biofilms Microbiomes. (2025) 11:59. doi: 10.1038/s41522-025-00695-w 40234406 PMC12000565

[30] Du W Jiang S Yin S Wang R Zhang C Yin BC . The microbiota-dependent tryptophan metabolite alleviates high-fat diet-induced insulin resistance through the hepatic AhR/TSC2/mTORC1 axis. PNAS. (2024) 121:e2400385121. doi: 10.1073/pnas.2400385121 39167602 PMC11363250

[31] Li X Zheng P Zou Y Guan L Li N Liu J . Dietary inulin ameliorates obesity-induced severe acute pancreatitis via gut-pancreas axis. Gut Microbes. (2024) 16:2436949. doi: 10.1080/19490976.2024.2436949 39653685 PMC11633219

[32] Xie J Du L Lu Y Guo X Zhou X Tong Y . Akkermansia muciniphila alleviates severe acute pancreatitis via Amuc1409-Ube2k-Foxp3 axis in regulatory T cells. Advanced Sci (Weinheim Baden-Wurttemberg Germany). 12(30):e04214. doi: 10.1002/advs.202504214 40464424 PMC12376690

[33] Feng Y Chen W Chen J Sun F Kong F Li L . Dietary emulsifier carboxymethylcellulose-induced gut dysbiosis and SCFA reduction aggravate acute pancreatitis through classical monocyte activation. Microbiome. (2025) 13:83. doi: 10.1186/s40168-025-02074-1 40128912 PMC11931840

[34] Facchin S Calgaro M Savarino EV . Rethinking short-chain fatty acids: A closer look at propionate in inflammation, metabolism, and mucosal homeostasis. Cells. (2025) 14:1130. doi: 10.3390/cells14151130 40801563 PMC12346497

[35] Wang Q Liu X Du Z Zheng Y Meng Z Lv Z . Astragalus polysaccharide reduces the severity of acute pancreatitis under a high-fat diet through enriching L. reuteri and propionate. Int J Biol Macromol. (2025) 298:140021. doi: 10.1016/j.ijbiomac.2025.140021 39837455

[36] Wang Y Wang X Chen Z Zheng J Liu X Zheng Y . Akkermansia muciniphila exacerbates acute radiation-induced intestinal injury by depleting mucin and enhancing inflammation. ISME J. (2025) 19:wraf084. doi: 10.1093/ismejo/wraf084 40305678 PMC12089034

[37] Wang L Zhang P Li C Xu F Chen J . A polysaccharide from <i>Rosa roxburghii</i> Tratt fruit attenuates high-fat diet-induced intestinal barrier dysfunction and inflammation in mice by modulating the gut microbiota. Food Funct. (2022) 13:530–47. doi: 10.1039/d1fo03190b 34932054

[38] Lee J Lim JW Kim H . Lycopene inhibits IL-6 expression by upregulating NQO1 and HO-1 via activation of Nrf2 in ethanol/lipopolysaccharide-stimulated pancreatic acinar cells. Antioxid (Basel Switzerland). (2022) 11:519. doi: 10.3390/antiox11030519 35326169 PMC8944646

[39] Zhang C Chen S Wang Z Zhang J Yu W Wang Y . Exploring the mechanism of intestinal bacterial translocation after severe acute pancreatitis: The role of Toll-like receptor 5. Gut Microbes. (2025) 17:2489768. doi: 10.1080/19490976.2025.2489768 40243695 PMC11980482

[40] Zhang T Zhao S Dong F Jia Y Chen X Sun Y . Novel insight into the mechanisms of neurotoxicity induced by 6:6 PFPiA through disturbing the gut-brain axis. Environ Sci Technol. (2023) 57:1028–38. doi: 10.1021/acs.est.2c04765 36594808

