# Supplementary material for: SCO‑792 alleviates HFD‑aggravated chronic pancreatitis by modulating the gut–pancreas axis via the AMPK/ACC and TLR4/NF‑κB signaling pathways
Source: Front Immunol. 2026 Sep 3;17:1923107. doi: 10.3389/fimmu.2026.1923107 (PMC13581505; doi:10.3389/fimmu.2026.1923107)
Supplement: Supplementary file 1 [file Table1.docx]

**Manuscript ID:** 1923107

**Title:** SCO-792 alleviates HFD-**aggravated** chronic pancreatitis by modulating the gut –pancreas axis via AMPK/ACC and TLR4/NF-κB Pathways

**Journal:** Frontiers in Immunology - Inflammation

Supplementary Material

**Supplementary Figure S1**

**Figure S1. Plasma lipase levels in experimental mice.** **Groups: NC, normal control group; HFD, high-fat diet control group; CAE,** caerulein-induced chronic pancreatitis model group with HFD feeding**; SCO-792, SCO-792 intervention group. Data are presented as mean ± SD, n = 6-8 mice per group. *****P* < 0.0001.**

**Supplementary Figure S2**

**Figure S2. SCFAs concentrations in** ***A. muciniphila* culture supernatants.** (A) PCA score plot showing separation of SCFAs profiles. (B) Quantitative comparison of individual SCFA levels between NC and AKK groups. NC, sterile blank medium supernatant; AKK, *A. muciniphila* culture supernatant. Data are shown as means ± SEM, n = 4 per group. Unpaired Student’s t-test. *P < 0.05, **P < 0.01; ns, not significant.

**Supplementary Figure S3**

**Supplementary Figure S3. In silico analyses for the interaction between propionate and enteropeptidase.** (A) Three‑dimensional structure of the propionate‑enteropeptidase docking complex. The enlarged panel illustrates the ligand‑binding pocket. (B) Two‑dimensional interaction plot showing critical amino‑acid residues participating in propionate binding. (C) Backbone RMSD of the propionate‑enteropeptidase complex throughout the 100 ns molecular dynamics simulation. (D) Residue‑specific root‑mean‑square fluctuation (RMSF). (E) Radius of gyration (Rg). (F) Solvent‑accessible surface area (SASA). (G) Time‑dependent count of hydrogen bonds formed between propionate and enteropeptidase. (H) Free‑energy landscape (FEL) analysis. (I) Per‑residue decomposition of binding free energy. (J) Electrostatic surface potential of enteropeptidase. (K) Structural snapshots of the complex obtained at 0 ns, 50 ns, and 100 ns during simulation.

**Supplementary Figure S4**

**Figure S4. Confirmation of A. muciniphila colonization in the gut.** Colonization of *A. muciniphila* was verified by qPCR analysis of fecal samples (n = 7 per group). The relative abundance of *A. muciniphila* was quantified using species‑specific primers and normalized to total bacterial 16S rRNA levels. *** p < 0.001.
